# Investigation of a Multi-Layer Absorber Exhibiting the Broadband and High Absorptivity in Red Light and Near-Infrared Region

**DOI:** 10.3390/nano13040766

**Published:** 2023-02-18

**Authors:** Guoxiang Peng, Wei-Zheng Li, Ling-Chieh Tseng, Cheng-Fu Yang

**Affiliations:** 1 School of Ocean Information Engineering, Jimei University, Xiamen 361021, China; 2 Department of Chemical and Materials Engineering, National University of Kaohsiung, Kaohsiung 811, Taiwan; 3 Department of Aeronautical Engineering, Chaoyang University of Technology, Taichung 413, Taiwan

**Keywords:** ultra-wideband, absorber, COMSOL Multiphysics^®^ software, localized surface plasmon resonance, Fabry-Perot cavity resonance

## Abstract

In this study, an absorber with the characteristics of high absorptivity and ultra-wideband (UWB), which was ranged from the visible light range and near-infrared band, was designed and numerically analyzed using COMSOL Multiphysics^®^ simulation software (version 6.0). The designed absorber was constructed by using two-layer square cubes stacked on the four-layer continuous plane films. The two-layer square cubes were titanium dioxide (TiO_2_) and titanium (Ti) (from top to bottom) and the four-layer continuous plane films were Poly(N-isopropylacrylamide) (PNIPAAm), Ti, silica (SiO_2_), and Ti. The analysis results showed that the first reason to cause the high absorptivity in UWB is the anti-reflection effect of top TiO_2_ layer. The second reason is that the three different resonances, including localized surface plasmon resonance, the propagating surface plasmon resonance, and the Fabry-Perot (FP) cavity resonance, are coexisted in the absorption peaks of the designed absorber and at least two of them can be excited at the same time. The third reason is that two FP resonant cavities were formed in the PNIPAAm and SiO_2_ dielectric layers. Because of the combination of the anti-reflection effect and the three different resonances, the designed absorber presented the properties of UWB and high absorptivity.

## 1. Introduction

A metamaterial absorber is a structure of multi-layer materials being used to enhance absorptivity or shield electromagnetic radiation. However, the metamaterials with different multi-layer structures have the advancement in material technologies and sciences. In addition, a metamaterial with multi-layer materials can have different important applications in different electromagnetic devices. For example, Sarto et al. investigated a one-dimensional photonic bandgap structure on plastic substrate, which could be applied to shield electromagnetic field in the radio frequency range [1]. Larciprete et al. investigated a one-dimensional and transparent metallodielectric photonic band gap to confine light and enhance the nonlinear response of the layers. In this structure, the incident light intensity had the property of a nonlinear transmission dependence [2]. Larciprete et al. also used magnetron sputtering to deposit the multi-layer Ag and Ta_2_O_5_ films, which could be used to investigate the property of its second order nonlinear optical response [3]. Hence, those metamaterials using different multi-layer materials with different stack formats and structures are used to design the absorbers with more benefits as compared with the conventional absorbers, such as enhanced effectiveness, wider adaptability, and further miniaturization. Intended applications for the metamaterial absorbers include sensors [4], photodetectors [5], infrared camouflage [6], thermophotovoltaics [7], solar photovoltaics [8,9], etc.

In the past, many technologies had been investigated to broaden the bandwidths and enhance the absorptivity of the designed absorbers, such as the effects of localized surface plasmon (LSP) resonance [10,11], (asymmetric) highly lossy Fabry–Perot (FP) cavity resonance [12,13], and propagating surface plasmon (PSP) resonance [11,14]. Therefore, many studies investigated the multi-layer absorbers by the combinations of the above technologies to achieve the absorbers with the ultra-wideband (UWB) in different wavelength ranges [11,12]. Poly(N-isopropylacrylamide) (PNIPAAm) is a temperature-responsive polymer, which can use the via free-radical polymerization to synthesize it from N-isopropylacrylamide, and PNIPAAm is useful in different applications after it is readily functionalized. For example, Mikulchyk et al. developed a new N-isopropylacrylamide-based photopolymer, which has lower toxicity than acrylamide-based photopolymers and is sensitive to temperature, as a temperature sensor for holographic recording in transmission and reflection modes [15]. Shoji et al. used the plasmonic optical trapping property of PNIPAAm microassembly to construct a highly sensitive detection of organic molecules [16]. They used the combination of an enhanced optical force and a photothermal effect to easily form the PNIPAAm microassemblies. They also showed that the PNIPAAm-based hydrogel could be utilized as a layer of stimuli-responsive insulator, which could enhance the sensing capability in the visible band and having the perfect absorption in near-infrared (NIR) region.

In the past, the polymer-based materials were used to investigate the absorber in the microwave range. For example, Lu et al. investigated a microwave absorber using the metal-lossless polyvinyl butyral-metal multilayers to absorb both transverse electric (TE) and transverse magnetic (TM) in the range of 3~24.5 GHz [17]. Choi et al. used a patterned resistive sheet, glass/epoxy, and glass/MWCNT-epoxy to form a multi-layer structure to design an absorber for X-band radar [18]. However, it is very difficult to find the use of polymer materials to investigate the optical absorbers in the range of visible light and NIR. The physical phenomena for PNIPAAm to have the highly sensitive detection of organic molecules are that it has the property of the strong LSP resonant excitation. Therefore, the PNIPAAm is selected as the dielectric material in the newly investigated absorber to achieve the properties of high absorptivity and UWB in the range of partial visible light (red light) and NIR. The first novelty is that the PNIPAAm film was used as the dielectric material between a square cube Ti layer and a Ti layer on the top of four-layer continuous plane films to construct an asymmetric FP resonant cavity. Many software packages have been used as the tools for numerical simulations to find and understand the mechanisms of absorption principles in their designed absorbers, such as finite difference time domain (FDTD) method [11,12] and Computer Simulation Technology Microwave Studio [19]. COMSOL Multiphysics^®^ is a simulation platform and it is one of the most frequently used numerical analysis software, which can be used to compute the optical properties of the designed nanophotonic devices [20]. COMSOL Multiphysics^®^ can also process all of the steps in the workflows of modeling and simulation, including of importing material properties and defining geometries, and the physics that describe specific phenomena to solve and postprocess the models for generating the accurate results.

In this study, a commercially available COMSOL Multiphysics^®^ simulation software was used as the tool to design and simulate the investigated novel absorber with the property of UWB. The designed absorber had the structures of four-layer continuous plane films and a two-layer square cubes stacked upper the four-layer films, which generates some important novelties in this designed multilayer structure. The second novelty is that the top TiO_2_ square cube is not only designed as an anti-reflective film and as an anti-corrosion layer for preventing the Ti square layer from being oxidized. Another important novelty is that the LSP resonance, PSP resonance, and the asymmetric FP cavity resonance were importantly excited in the investigated absorber at the same time to reach the high absorptivity in an UWB. The third novelty is that is that two FP resonant cavities were formed in the PNIPAAm and SiO_2_ dielectric layers. Thus, the simulation results would show that the proposed innovate structure had the maximum absorptivity of 99.9% and could achieve a high absorptivity of 90% and a high average absorptivity of 97.9% in the wide wavelength range of 620 to 3590 nm. Because the effect of high absorptivity and UWB, the absorber proposed in this paper have great prospects in the fields of perfect cloaking, solar power generation, and thermal electronic equipment.

## 2. Experimental Procedures

**Figure 1:**
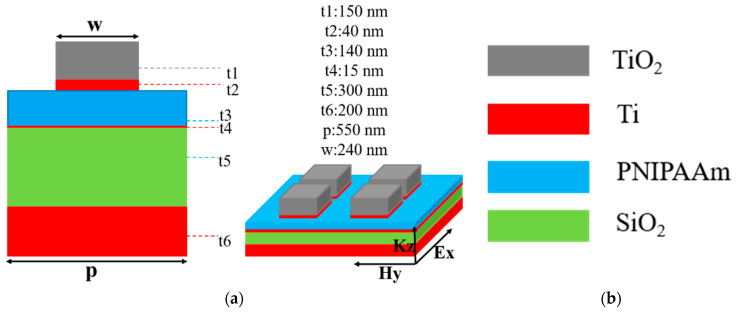
(**a**) Side view for the unit cell of the designed UWB absorber and the stereogram image represented a small region of the designed UWB absorber consisting of the symmetric arrangements of a two-layer square cubes stacking on the four-layer continuous plane films, (**b**) the colors used to represent different materials.

The related geometric structure of the investigated absorber is presented in Figure 1, in which, the thicknesses of t1 (titanium dioxide, TiO_2_), t2 (titanium, Ti), t3 (Poly(N-isopropylacrylamide, PNIPAAm), t4 (Ti), t5 (silica, SiO_2_), and t6 (Ti) were 150 nm, 40 nm, 140 nm, 15 nm, 300 nm, and 150 nm, respectively, and the reasons to define the optimum lengths for each layer were shown in next section. For the four-layer square films and the two-layer square cubes, their side lengths were 550 nm and 240 nm.

In this study, in order to find the optimal structure parameters for the designed absorber having the properties of the high absorptivity in an UWB, the investigated device’s structure was numerically simulated and analyzed to demonstrate and optimize using the COMSOL Multiphysics^®^ simulation software (version 6.0, manufacturer: COMSOL Multiphysics^®^, Stockholm, Sweden; version 6.0) The COMSOL Multiphysics^®^ is one of the most frequently used numerical analysis software for computing the optical properties in a multi-layer structure. The accuracy and reliability of the numerical analysis results of COMSOL Multiphysics^®^ were ensured by many researchers [20,21]. Additionally, COMSOL Multiphysics^®^ has a built-in Model Builder software, which has the property parameters of many built-in materials and can provide a single-physics modeling capability for fully coupled Multiphysics. Therefore, the Model Builder software built-in COMSOL Multiphysics^®^ was used to construct the gold-standard models of the investigated nanophotonic devices, the COMSOL Multiphysics^®^ was used to simulate the designed devices. An ideally investigated multi-layers were stacked in the *z*-axis direction, and the period boundary conditions were set in *x*-axis and *y*-axis directions. A plane wave with TM polarization and the electric and magnetic fields, which was polarized in the directions of *x*-axis and *y*-axis, respectively, was used as the normally incident source onto the investigated absorber.

**Figure 2:**
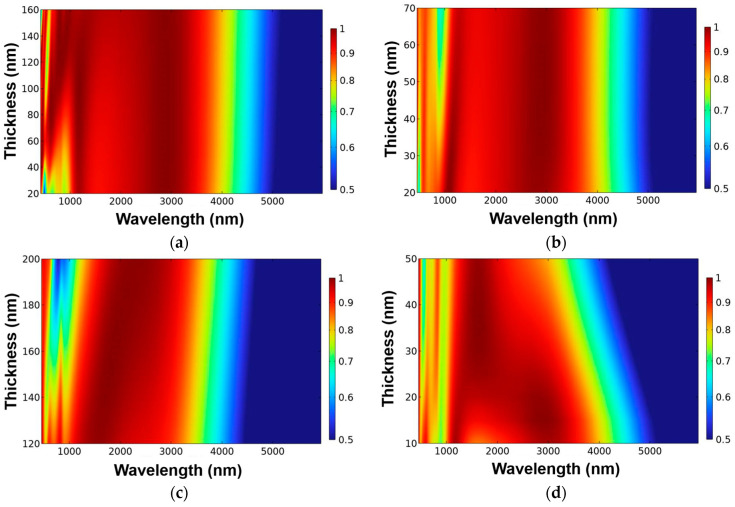

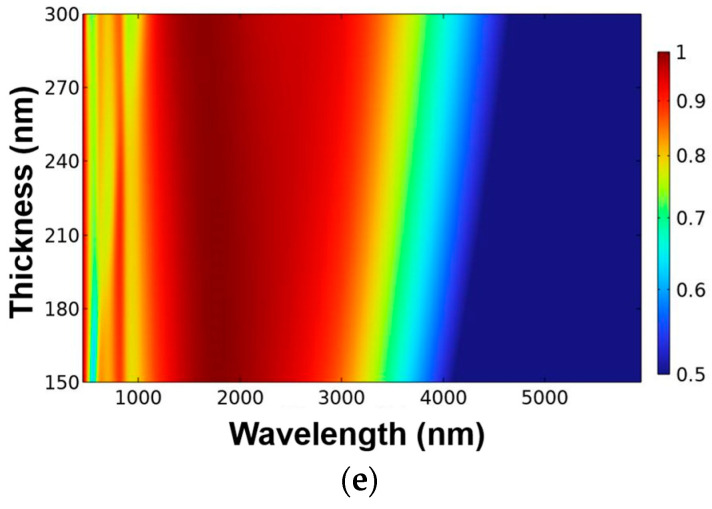
Effects of different thicknesses on the absorptivity of each layer of the designed absorber (**a**) anti-reflection TiO_2_ layer (t1), (**b**) upper Ti layer (t2), (**c**) PNIPAAm layer (t3), (**d**) lower Ti layer (t4), and (**e**) SiO_2_ dielectric layer (t5).

In order to demonstrate that each layer of material in our proposed structure is designed in its optimization thickness, the thickness of each layer material is changed to find its optimal thickness at when the thicknesses of other layers are not changed. Figure 2 illustrates the variations of absorptivity for the different layers of the designed absorber at when the thickness of each-layer material was changed, and that is the basis method to determine the optimization parameters of the investigated absorber with broadband and high absorptivity. Figure 2 illustrates the variations of absorptivity of the designed absorber caused by only changing the thickness of one layer but keeping other layers being unchanged for the designed structure presented in Figure 1. The wavelength used to find the optimization thickness ranged from 600 to 6000 nm, but even the same material was used, the thickness to be used in simulation was different. The results in Figure 2 show that the effects of thicknesses of the different layers on the numerical variations of the absorptivity were apparently observed.

Figure 2a illustrates the variations of absorptivity of the designed absorber caused by only changing the thickness of the TiO_2_ anti-reflection layer (t1) from 20 to 160 nm for the designed structure presented in Figure 1. As Figure 2a shows, when t1 value was thinner than 100 nm, the lower absorptivity was presented in the range of 600~1050 nm. When t1 value was about 150 nm, the designed absorber had a high absorptivity in the range of 750~3800 nm, therefore, 150 nm was chosen as the thickness of TiO_2_ anti-reflection layer. Figure 2b–e illustrates the variations of absorptivity of the designed absorber caused by only changing the thicknesses of the upper Ti layer (changed from 20 to 70 nm), the PNIPAAm layer (changed from 120 to 200 nm), the lower Ti layer (changed from 10 to 50 nm), and the SiO_2_ dielectric layer (changed from 150 to 300 nm), respectively. As Figure 2b–e show, when their thicknesses were 150 nm, 40 nm, 140 nm, 15 nm, 300 nm, and 150 nm, the designed absorber illustrated the high absorptivity in the range of 750~3800 nm. Therefore, the thicknesses of 150 nm, 40 nm, 140 nm, 15 nm, 300 nm, and 150 nm were chosen as the optimization thicknesses of t1 (TiO_2_), t2 (Ti), t3 (PNIPAAm), t4 (Ti), t5 (SiO_2_), and t6 (Ti) layers, respectively.

## 3. Results

**Figure 3:**
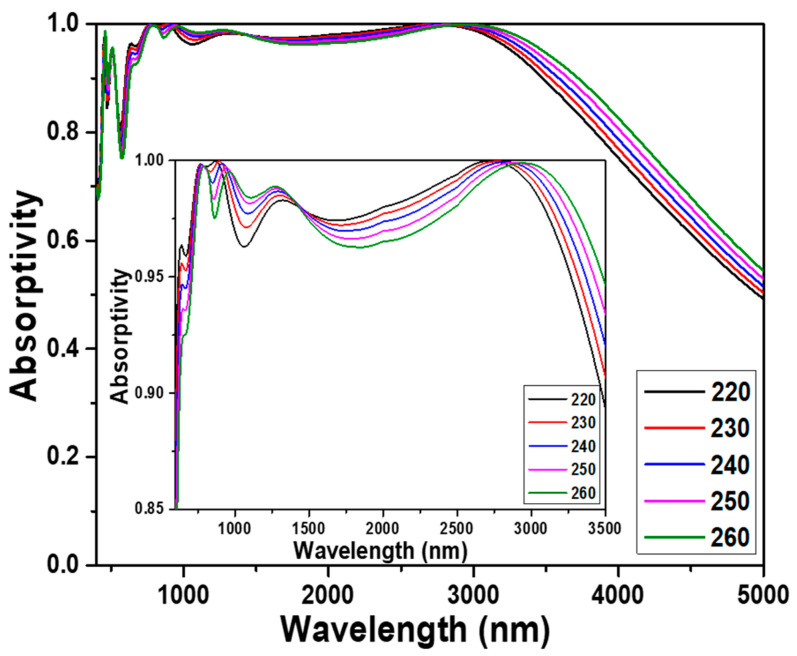
Effect of the side length of the two-layer square cubes on the absorption properties of the designed absorber.

First, the side length of the designed square cubes was changed from 220 to 260 nm to conduct the simulations at 10 nm intervals to prove that the 240 nm was the optimal length, and the comparisons of the simulation results are shown in Figure 3. As Figure 3 shows, the absorption peaks located at about 470, 510, 780, 940, 1280, and 2880 nm were presented in the absorption spectrum of the investigated absorber. Thus, as the cube lengths were 220, 230, 240, 250, and 260 nm, the absorptivity to be higher than 90.0% was in the ranges of 600 to 3510 nm, 610 to 3550 nm, 620 to 3590 nm, 620 to 3660 nm, and 630 to 3730 nm. These results showed that as the side length of the square cubes increased from 220 to 260 nm, the start wavelength for the absorptivity to be higher than 90.0% was slightly shifted from 220 to 260 nm and the end wavelength with absorptivity to be higher than 90.0% was shifted from 3510 to 3730 nm. Figure 3 also shows that these absorption peaks in the range of 2500~3200 nm had the absorptivity to be higher than 99.0% and had the maximum absorptivity of 99.9%, which were in the ranges of 2370 to 2980 nm, 2450 to 3040 nm, 2520 to 3080 nm, 2580 to 3130 nm, and 2640 to 3190 nm as the cube lengths were 220, 230, 240, 250, and 260 nm, respectively. Three main resonances are investigated and used to enhance the absorptivity of the designed absorbers including of the LSP resonance, the PSP resonance, and the FP cavity resonance.

An LSP resonance is the result of the generation of a surface plasmon in a thin film with thickness or a nanoparticle with the size smaller than or comparable to the wavelength of light used to excite the plasmon. A PSP resonance is generated by that when the incident light is used to stimulate on the surfaces of the positive and negative permittivity materials to cause the resonant oscillation of conduction electrons at the interface. Both LSP resonance and PSP resonance are belong to surface plasmon polarization (or resonance), but LSP resonance is different from PSP resonance. For PSP resonance the electron clouds oscillate collectively, and for LSP resonance the surface plasmon propagates back and forth between the two ends of the designed structure. The waves created in PSP resonance can also be tuned by changing the geometry of the metal nanostructure. The absorption peaks in the range of 2500~3200 nm had an apparent red shift, which were shifted from 2730 nm to 2950 nm as the side length of the square cube increased from 220 nm to 260 nm. However, the absorption peaks of 470, 510, 780, 940, and 1280 nm had no the property of red shift as the side length of the square cubes increased.

These results prove that this absorption peak located in the range of 2500~3200 nm is caused by the PSP resonance, which is the first reason to make the designed UWB absorber having high absorptivity in a wideband range. The average absorptivity is calculated by the equation, where *R* = $\int_{\lambda_{1}}^{\lambda_{2}}$ *R*(*λ*)*dλ*/(*λ*_2_ − *λ*_1_), where *λ*_1_ and *λ*_2_ are the start and end wavelengths of the spectra with absorptivity higher than 90.0%. When 600, 610, 620, 620, and 630 nm were used as *λ*_1_ and 3510, 3550, 3590, 3660, and 3730 nm were used as *λ*_2_, the average absorptivity reached the high values of 97.7%, 97.7%, 97.9%, 97.5%, and 97.4%, respectively. The calculation results of the designed absorber presents the high absorptivity in a broadband range, therefore, this result match the results shown in Figure 2. Even the thickness of the each-layer material is changed, the designed absorber has the high absorptivity in a broadband range. The inset diagram in Figure 3 also shows that even the absorptivity in the range of about 1350~2850 nm decreased as the cube length decreased from 220 to 260 nm, thus the absorptivity in the out the range of 1350~2850 nm increased from 220 to 260 nm. When the cube length was 240 nm, the designed novel absorber had the highest average absorptivity in the range of absorptivity higher than 90%, therefore, it was used as the optimum length for further analyses and designs.

**Figure 4:**
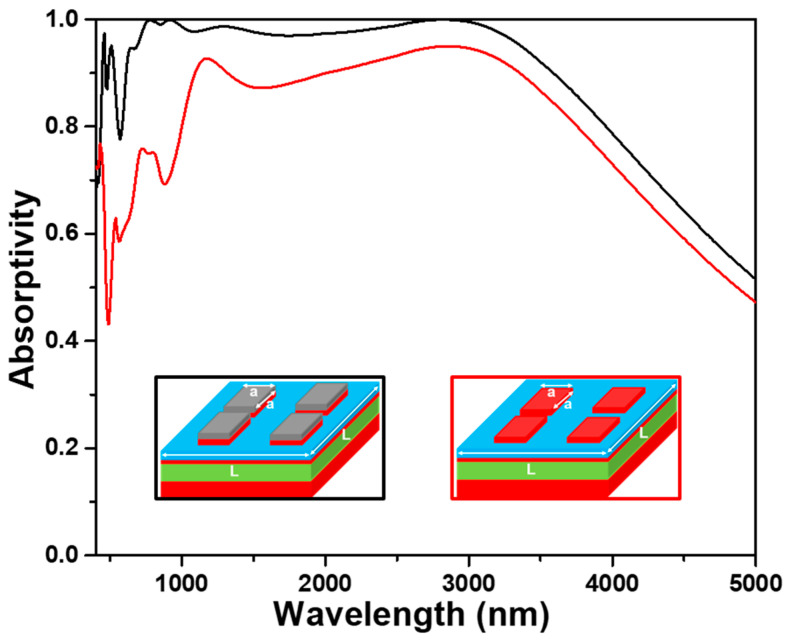
Effect of the TiO_2_ anti-reflection layer on the absorption properties of the designed absorber. Black line and red line were used for with and without anti-reflection layer.

The absorption spectra of the investigated absorber with and without the upper TiO_2_ layer (t1 layer) under normal incident light were numerically simulated to prove the anti-reflection effect of the upper TiO_2_ layer, and the results are shown in Figure 4. Apparently, the absorption spectrum for the absorber with the upper TiO_2_ layer had the absorption peaks located at 470, 510, 780, 940, 1280, and 2880 nm and absorption spectrum. In Figure 4, the absorber with the anti-reflection TiO_2_ layer has a superior absorptivity as compared to the absorber without anti-reflection TiO_2_ layer in the region of visible light. For the investigated absorber with the anti-reflection TiO_2_ layer, the absorptivity was higher than 90.0% in the range of 620~3590 nm and the absorptivity was higher than 96.5% in the range of 715~3340 nm. It also shows that the absorptivity for peaks located at 780, 940, and 2880 nm is higher than 99%, it means that the investigated absorber with the anti-reflection TiO_2_ layer presents the perfect absorption property at those wavelengths. For the investigated absorber without the anti-reflection TiO_2_ layer, the maximum absorptivity was 95.0%, and the absorptivity to be higher than 90.0% only presented in a small range. These comparison results also prove that the topmost TiO_2_ cube layer can act as an antireflection layer to enhance the absorptivity in a wide wavelength range. When TiO_2_ cube on the topmost layer is used as anti-reflection layer, the simulation results are as expected, the absorptivity of the absorber in the whole simulation region (400~5000 nm) is higher than absorber without the TiO_2_ anti-reflection layer. The results further prove that the second reason to cause the investigated absorber having high absorptivity in the broadband region is caused by the addition of a TiO_2_ anti-reflection layer.

**Figure 5:**
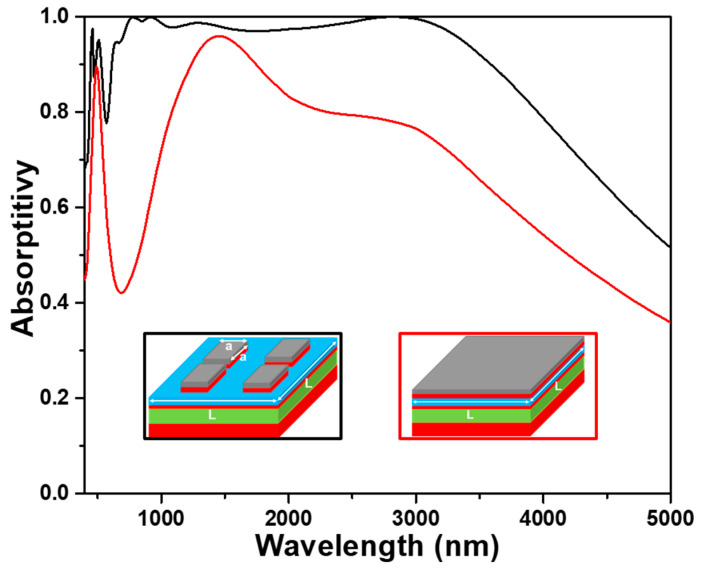
Effect of the TiO_2_ and Ti layers with different structures on the absorption properties of the designed absorber. Black line for TiO_2_ (t1) and Ti (t2) layers with a square length of 240 nm and red line for and TiO_2_ (t1) and Ti (t2) layers with full planes (550 nm).

In order to verify the advantages of the investigated novel structure of a two-layer square cubes stacked on the four-layer square films, an absorber with the structure of the six-layer continuous plane films, as the structure shown in the inset of Figure 5, was also simulated. The thicknesses of each layer were the same with those of the proposed absorber shown in Figure 1, and both the absorption spectra are compared in Figure 5 for the two different devices shown in the insets of Figure 5. As Figure 5 shows, one sharp absorption peak and one broaden absorption peak, which were located at about 490 and 1450 nm, and one unapparent transition point, which was in the range of 2900~3050 nm, was revealed in the absorber with the six-layer continuous plane films. However, the absorption peaks located at 470, 510, 780, 940, and 1280 nm in the spectrum of our designed absorber were not observed in the device with the six-layer continuous plane films. The structure with the six-layer continuous plane films had a maximum absorptivity of 95.9% at 1450 nm and the absorptivity higher than 90% was only in the range of 1230~1760 nm. The combination effect of LSP resonance and PSP resonance generated on the TiO_2_-Ti films of the six-layer continuous plane films is thought as the reason to cause this result. However, this device does not reach the characteristics of ultra-wideband and high absorptivity.

A FP etalon or interferometer is an optical cavity which can be constructed using two thin mirrors (metals) to form the parallel reflecting surfaces. The two thin mirrors are separated by a lossless dielectric film with the thickness of at least a quarter-wavelength of the incident light. For that, as Figure 1 shows, a FP cavity resonance can be formed by the Ti square cube (t2) and Ti plane film layer (t4) separated by PNIPAAm layer and another FP cavity resonance can be formed by the two Ti plane film layers (t4 and t6) separated by SiO_2_ layer. The results in later section will further prove that TiO_2_ (t1)-Ti (t2) square cubes can cause the excitation of the LSP resonance and the Ti (t2)-PNIPAAm (t3)-Ti (t4)-SiO_2_ (t5)-Ti (t6) layers can cause the excitation of the LSP resonance and the FP cavity resonance in the PNIPAAm and SiO_2_ layers. This comparison result further proves that the investigated structure can excite the multiple electromagnetic resonance modes at the same time. Therefore, the absorptivity and average absorptivity of the proposed absorber are much better than those of absorber with the six-layer continuous plane films due to the enhancements of the synergy resonances of the LSP and FP cavity in the red light and NIR band of analyzed wavelength.

**Figure 6:**
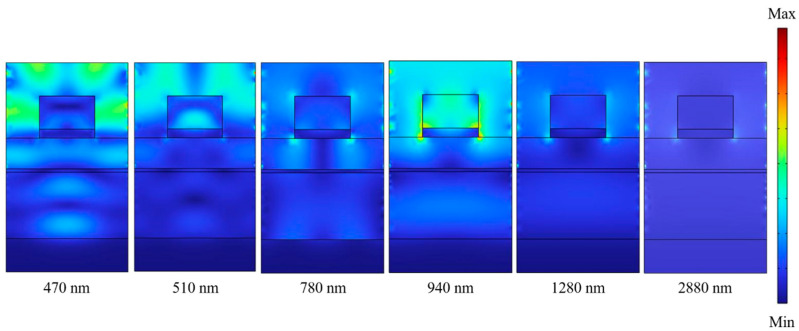
Distributions of electric field intensities under normal incident TE polarized light with different exciting wavelengths.

**Figure 7:**
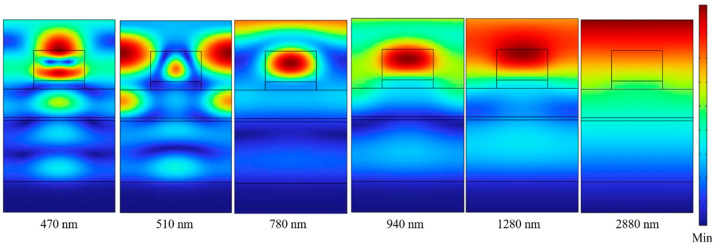
Distributions of magnetic field intensities under normal incident TE polarized light with different exciting wavelengths.

In order to prove the physical mechanisms exciting in the investigated absorber to cause the properties of high absorptivity and UWB, the distributions of electric field intensities (|E|) and those of magnetic field intensities (|H|) in the x-z plane are simulated under the different wavelengths and the results are shown in Figure 6 and Figure 7, respectively. The TE-polarized light at the normal direction with the exciting wavelengths of 470, 510, 780, 940, 1280, and 2880 nm were used as the incident light to excite the investigated absorber, because these absorption peaks are presented in the absorption spectrum shown in Figure 5. When an incident light is used to excite on the surfaces of the successive positive and negative thin-film permittivity materials, the PSP resonance generated by the resonant oscillations of conduction electrons will be generated between their interface. The distributions of electric field intensities under the normal incident TE polarized lights of different wavelengths, as Figure 6 shows, were coupled around the edge of the first TiO_2_ layer and were positioned in the gaps of the two-layer square cubes and penetrated through the PNIPAAm layer and into the SiO_2_ layer and they were reflected by the bottom Ti layer. These results prove that the different surface plasmon resonances are clearly excited in the investigated absorber.

Figure 6 shows that the strong electric fields not only existed on the top of TiO_2_-Ti cubes and below the four continuous films. As the wavelengths of incident light were 470, 510, 780, 940, 1280, and 2880 nm, the results in Figure 6 really reveal that the electric fields were mainly distributed at the edges and the internals of the top TiO_2_-Ti cubes. When the wavelengths of incident light were 470, 510, 780, 940, and 1280 nm, the electric fields were also distributed in the internals of the PNIPAAm and the SiO_2_ layers. An LSP resonance is generated as the wavelength of exciting light is comparable to or longer than the confinement of a surface plasmon in a nanoparticle. For that, as the wavelength of exciting light is longer than 250 nm, the rectangular cubes of the two-layer TiO_2_ and Ti square cubes can generate the LSP resonance. However, the excitation intensity of the LSP resonance depends on the wavelength of incident light and the sizes of the designed nano-structure or the nanoparticle materials. As the wavelengths of incident light were 470, 510, 780, 940, and 1280 nm, as Figure 6 shows, strong electric field appeared in the area adjacent to the TiO_2_ and Ti square cubes. These phenomena indicate that the high absorptivity at the incident lights of 470, 510, 780, 940, and 1280 nm can be attributed to the excitations of different plasmon resonances at the investigated absorber. The first is the LSP resonance between the top TiO_2_-Ti cubes layers and metal Ti film and second is the FP cavity resonance in the PNIPAAm and SiO_2_ layers.

In addition, the magnetic field distributions for the exciting wavelengths of 470, 510, 780, 940, 1280, and 2880 nm are shown in Figure 7. The results in Figure 7 have clearly shown that the magnetic fields with different exciting wavelengths were distributed in the TiO_2_ and Ti square cubes and in the PNIPAAm and SiO_2_ dielectric layers. The FP cavity resonance in an absorber can be generated by two parallel flat semi-transparent metals as the reflective mirrors. In the investigated absorber, the Ti layers of t2, t4, and t6 are separated by the lossless PNIPAAm and SiO_2_ dielectric layers. The PNIPAAm and SiO_2_ layers are used as the lossless dielectric layers, therefore, the t2-t4 and t4-t6 Ti metal layers separated by PNIPAAm and SiO_2_ layers have formed two FP cavities. Because the exciting wavelengths of 470, 510, 780, 940, and 1280 nm satisfy the condition of the FP cavity resonance, the normal incident light will conduct the constructive or destructive interferences with the reflected light. Therefore, the magnetic-distribution patterns really show that the FP cavity resonance is really excited in PNIPAAm and SiO_2_ dielectric layers.

Figure 7 also shows that as the exciting wavelengths are different, the intensities of the magnetic distributions caused by the FP cavity resonances in the cavity layers of PNIPAAm and SiO_2_ were different. The magnetic-distribution patterns in Figure 7 show that as the exciting wavelengths were 470, 510, 780, 940, and 1280 nm, the LSP resonance is also excited in the TiO_2_-Ti cubes. Because the incident light was reflected between the t2-t4 metal layers for the PNIPAAm dielectric layer, there was also a strong magnetic field distribution between the t4-t6 metal layers for the SiO_2_ dielectric layer. Therefore, the perfect absorptivity at the wavelengths of around 780 and 940 nm are caused by the combinations of the LSP and FP cavity resonances. As the wavelength of normal incident light was 2880 nm, the electric field was distributed at the around of the two-layer square cubes and the magnetic field was distributed in the whole region of the investigated absorber. Therefore, the perfect absorptivity at the wavelength of around 2880 nm is mainly caused by the PSP resonance in the whole device. These results further prove that the strong absorption in the broadband region is caused by the combination of effects of the LSP, PSP, and FP cavity resonances.

**Figure 8:**
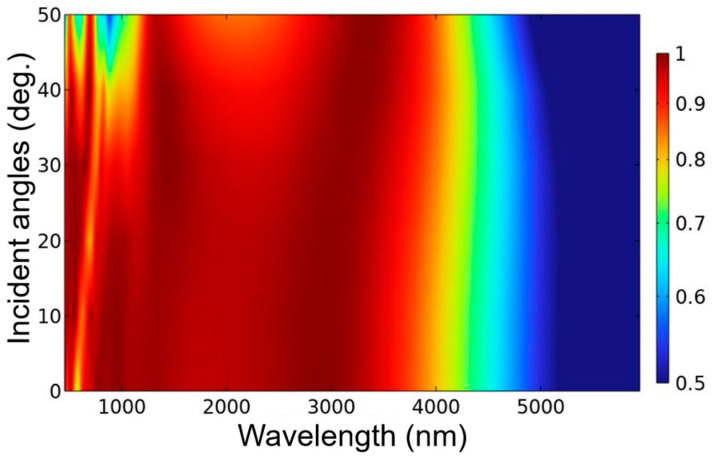
Evolution absorptivity of TE-polarized light with normal direction and different oblique incidence angles.

The calculation results show that the absorption properties of the investigated absorber are much better than those of the absorber with the six-layer continuous plane films because of the enhancements of the LSP and FP cavity resonances in the range of the analysis wavelength. The evolution absorption performances of the incident light with the angles of 0~50° at TE polarization is shown in Figure 8. When the investigated was excited under the TE polarization and the angles of incident light were in the range of 0~32°, it was clear that the absorber was able to absorb more than 90% in the spectral range of about 900~3500 nm. When the incident light had a larger angle of 40°, the designed absorber had the absorptivity of more than 90% in the range of about 1150~3500 nm, with a broad bandwidth of 2350 nm. When the incident light had a larger angle of 50°, the designed absorber had the absorptivity of more than 80% in the range of about 1200~3600 nm, with a broad bandwidth of 2400 nm. These results suggest that the designed absorber has the property of incident-angle insensitive. In addition, the good absorption performance of the designed absorber is also polarization-independence because of its high symmetrical structure.

**Figure 9:**
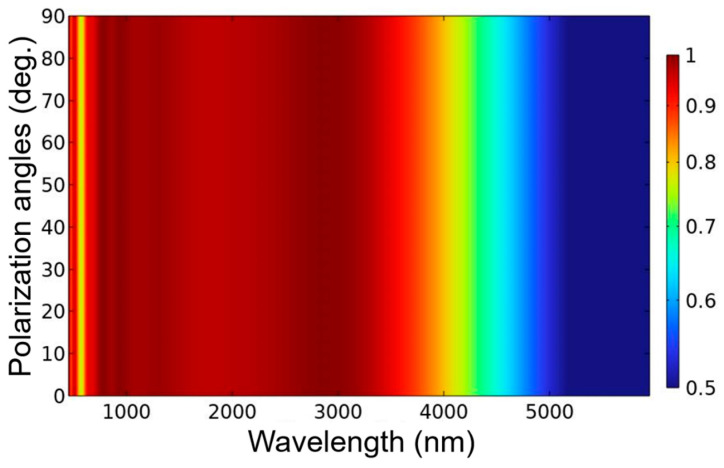
Evolution absorptivity of TE-polarized light with normally incident light in different polarization angle.

The effect of different polarization angles on the absorption performances of the designed absorber with normal incident light is shown in Figure 9. A polarization angle of 0° indicates that the polarization direction of the incident light is along in the x direction (or called as TE polarization), and a polarization angle of 90° indicates that the polarization direction of incident light is along in the Y direction (or called as TM polarization). In Figure 9, the distributions for evolution absorptivity show that the absorptivity is almost unchanged as polarization angles increased from 0° to 90°, which means that the absorption performance had no change with the variation of the polarization angles. Polarization-independent means that the investigated absorber has high absorption efficiency (high absorptivity), that makes the investigated absorber having more valuable in the practical applications. To the best of our knowledge, these numerical analyses have proven that our investigated absorber has the superior performances because it has the properties of high absorptivity in the broadband range of 625~3290 nm (red light and near-infrared region), polarization-independent, and wide-angle in the range of 0~32°. In the past, Alici et al. investigated a resonant absorber with broadband at the near-infrared band, but its bandwidth (absorptivity higher than 90%) was 825 nm (ranged from about 1080 to 1905 nm) [21]. Ding et al. used the highly lossy metals to design the broadband near-infrared metamaterial absorbers, and the bandwidth of the optimally investigated absorber was about 1000 nm (800~1800 nm) [22]. Zhang et al. designed a broadband near-infrared absorber based on an all-metallic metasurface, the bandwidth of the optimally investigated absorber was about 1500 nm (900~2400 nm) [23]. However, the investigated broadband absorber had a broad bandwidth of about 2665 nm (625~3290 nm), which had wider absorption band and expanded from the red light to the near-infrared region. The investigated absorber provides a superior performance, including of wider high absorption band and higher absorptivity as compared with the most previously reported absorbers.

## 4. Conclusions

In this study, the influences of different structure parameters and geometries on the absorption performances of an investigated absorber were systematically analyzed through the simulation results. The absorption peaks of 470, 510, 780, 940, 1280, and 2880 nm were presented in the absorption spectrum of the investigated absorber. The absorption peak in the range of 2500~3200 nm had an apparent red shift from 2730 nm to 2950 nm as the side length of the square cube increased from 220 nm to 260 nm, which proves that this absorption peak located is caused by the PSP resonance. The effects of the antireflection TiO_2_ cube layer and the PSP, LSP, and FP cavity resonance were all designed in the investigated absorber, which made the UWB and multi absorption peaks to cause the high absorptivity possible. The numerical analysis results showed that the designed UWB absorber had the absorptivity of higher than 90% and a high average absorptivity of 97.9% in the range of 610~3600 nm, and the maximum absorptivity was 99.9%. For TE and TM polarized light with incident angle of 90°, the absorptivity in the designed bandwidth was higher than 90%. However, the investigated absorber had the advantages of ultra-wideband, simple structure, and polarization-insensitive.

## Data Availability

Not applicable.

## References

[1] Sarto M.S., Sarto F., Larciprete M.C., Scalora M., D’Amore M., Sibilia C., Bertolotti M. (2003). Nanotechnology of Transparent Metals for Radio Frequency Electromagnetic Shielding. IEEE Trans. Electromagn. Compat..

[2] Larciprete M.C., Sibilia C., Paoloni S., Bertolotti M., Sarto F., Scalora M. (2003). Accessing the optical limiting properties of metallo-dielectric photonic band gap structures. J. Appl. Phys..

[3] Larciprete M.C., Belardini A., Cappeddu M.G., De Ceglia D., Centini M., Fazio E., Sibilia C., Bloemer M.J., Scalora M. (2008). Second-harmonic generation from metallodielectric multilayer photonic-band-gap structures. Phys. Rev. A.

[4] Ferreira C., Ventura P., Grinde C., Morais R., Valente A., Neves C., Reis M. (2010). Characterization and testing of a shock absorber embedded sensor. Procedia Eng..

[5] Mokkapati S., Saxena D., Tan H.H., Jagadish C. (2015). Optical design of nanowire absorbers for wavelength selective photodetectors. Sci. Rep..

[6] Chandra S., Franklin D., Cozart J., Safaei A., Chanda D. (2018). Adaptive Multispectral Infrared Camouflage. ACS Photonics.

[7] Tong J.K., Hsu W.C., Huang Y., Boriskina S.V., Chen G. (2015). Thin-film ‘Thermal Well’ Emitters and Absorbers for High-Efficiency Thermophotovoltaics. Sci. Rep..

[8] Vora A., Gwamuri J., Pala N., Kulkarni A., Pearce J.M., Güney D.Ö. (2014). Exchanging Ohmic Losses in Metamaterial Absorbers with Useful Optical Absorption for Photovoltaics. Sci. Rep..

[9] Choi J.W., Shin B., Gorai P., Hoye R.L.Z., Palgrave R. (2022). Emerging Earth-Abundant Solar Absorbers. ACS Energy Lett..

[10] Agrawal A., Cho S.H., Zandi O., Ghosh S., Johns R.W., Milliron D.J. (2018). Localized Surface Plasmon Resonance in Semiconductor Nanocrystals. Chem. Rev..

[11] Liu J., Ma W.Z., Chen W., Yu G.X., Chen Y.S., Deng X.C., Yang C.F. (2020). Numerical analysis of a novel ultra-wideband metamaterial absorber with high absorptivity from visible light to near-infrared. Opt. Express.

[12] Chen W., Liu J., Ma W., Yu G.X., Chen J.Q., Cai H., Yang C.F. (2020). Numerical Study of Multilayer Planar Film Structures for Ideal Absorption in the Entire Solar Spectrum. Appl. Sci..

[13] Gevorgyan A.H., Golik S.S., Vanyushkin N.A., Efimov I.M. (2022). Asymmetric absorption in asymmetric dielectric Fabry-Perot resonator with cholesteric liquid crystal layer inside. Opt. Mater..

[14] Zhou Y., Qin Z., Liang Z., Meng D., Xu H., Smith D.R., Liu Y. (2021). Ultra-broadband metamaterial absorbers from long to very long infrared regime. Light Sci. Appl..

[15] Mikulchyk T., Martin S., Naydenova I. (2017). N-isopropylacrylamide-based photopolymer for holographic recording of thermosensitive transmission and reflection gratings. Appl. Opt..

[16] Shoji T., Sugo D., Nagasawa F., Murakoshi K., Kitamura N., Tsuboi Y. (2017). Highly Sensitive Detection of Organic Molecules on the Basis of a Poly(N-isopropylacrylamide) Microassembly Formed by Plasmonic Optical Trapping. Anal. Chem..

[17] Lu X., Chen J., Peng Z., Wu Z., Zhang A. An optically transparent and ultra-wideband absorber based on multi-layer structure. Proceedings of the 2019 Cross Strait Quad-Regional Radio Science and Wireless Technology Conference (CSQRWC).

[18] Kumar R., Kumar P. (2022). Fabry–Pérot cavity resonance based metamaterial absorber for refractive index sensor at infrared frequencies. Opt. Commun..

[19] Xu J., Lin C.H., Chen Y.T., Tseng H.W., Wang P., Yang C.F., Lin C.L. (2021). Design and Optimize a Red Color Reflector Using a Simulation Software. Sens. Mater..

[20] Weng C.E., Yang C.F., Chen Y.T. (2022). Effect of Different Stacking Order of Ta_2_O_5_ and SiO_2_ Films on the Reflective Properties of a Blue Distributed Bragg Reflector. Mod. Phys. Lett. B.

[21] Alici K.B., Turhan A.B., Costas M., Soukoulis C.M., Ozbay E. (2011). Optically thin composite resonant absorber at the near-infrared band: A polarization independent and spectrally broadband configuration. Opt. Express.

[22] Ding F., Dai J., Chen Y., Zhu J., Jin Y., Bozhevolnyi S.I. (2016). Broadband near-infrared metamaterial absorbers utilizing highly lossy metals. Sci. Rep..

[23] Zhang K., Deng R., Song L., Zhang T. (2019). Broadband Near-Infrared Absorber Based on All Metallic Metasurface. Materials.

